# Endoplasmic Reticulum Proteins Impact Penetrance in a *Pink1*-Mutant *Drosophila* Model

**DOI:** 10.3390/ijms26030979

**Published:** 2025-01-24

**Authors:** Melissa Vos, Fabian Ott, Hawwi Gillo, Giuliana Cesare, Sophie Misera, Hauke Busch, Christine Klein

**Affiliations:** 1 Institute of Neurogenetics, University of Luebeck, UKSH (Universitätsklinikum Schleswig-Holstein, Campus Lübeck), Ratzeburger Allee 160, Building 67 (BMF), 23562 Luebeck, Germany; 2 Medical Systems Biology Division, Lübeck Institute of Experimental Dermatology, University of Luebeck, 23562 Luebeck, Germany

**Keywords:** Parkinson’s disease, reduced penetrance, Pink1, *Drosophila melanogaster*, endoplasmic reticulum

## Abstract

Parkinson’s disease (PD) is a neurodegenerative disorder with a high variability of age at onset, disease severity, and progression. This suggests that other factors, including genetic, environmental, or biological factors, are at play in PD. The loss of PINK1 causes a recessive form of PD and is typically fully penetrant; however, it features a wide range in disease onset, further supporting the existence of protective factors, endogenous or exogenous, to play a role. The loss of Pink1 in *Drosophila melanogaster* results in locomotion deficits, also observed in PINK1-related PD in humans. In flies, Pink1 deficiency induces defects in the ability to fly; nonetheless, around ten percent of the mutant flies are still capable of flying, indicating that advantageous factors affecting penetrance also exist in flies. Here, we aimed to identify the mechanisms underlying this reduced penetrance in Pink1-deficient flies. We performed genetic screening in *pink1*-mutant flies to identify RNA expression alterations affecting the flying ability. The most important biological processes involved were transcriptional and translational activities, endoplasmic reticulum (ER) regulation, and flagellated movement and microtubule organization. We validated two ER-related proteins, zonda and windbeutel, to positively affect the flying ability of Pink1-deficient flies. Thus, our data suggest that these processes are involved in the reduced penetrance and that influencing them may be beneficial for Pink1 deficiency.

## 1. Introduction

Parkinson’s disease (PD) features a wide variability in disease signs and symptoms, severity, progression, and age at onset [1], suggesting elements exist that alter disease development. Furthermore, the identification of a surprisingly large number of carriers of allegedly pathogenic variants remaining free of disease or developing a mild form indicates the existence of reduced penetrance [2]. The best-studied example in the field of PD is LRRK2-linked PD: the autosomal dominant PD-causing mutation G2019S in *LRRK2* is the most commonly known genetic cause of PD; however, it has a low penetrance that is affected by environmental and genetic factors [3,4,5,6,7]. In this manuscript, we focus on the mechanisms of Pink1-related penetrance on the basis of unique findings in a Pink1 *Drosophila* model [8]. The recessively inherited forms of PD, caused by mutations in *pink1* and *Parkin,* are typically highly (to fully) penetrant, yet they come with an extensive range of ages at onset, supporting external factors to be pivotal [3,9]. For instance, studies show that smoking or caffeine intake reduces the risk of PD [10,11]. Furthermore, the progression of PD can be delayed with a protective diet, such as the Mediterranean diet, combined with physical exercise [12,13], further underscoring the importance of external factors in the progression of PD.

Several mechanisms were found to be linked to PD. One of the more recent findings is the impact of the ‘gut-brain’ axis on PD [14] (cite a review article here?). In PD patients, gut bacteria appear to be pro-inflammatory via modulation of lipid metabolism that, in turn, may induce neuroinflammation [14,15]. Interestingly, infection of the intestines exacerbates PD-like signs in Pink1-deficient mice [16], reinforcing the importance of the gut–brain axis. Of further note, PINK1 is a mitochondrial protein that also localizes to the ER–mitochondrial contact sites [17,18,19]. These ER–mitochondrial contact sites play an important role in calcium homeostasis, mitophagy, and lipid metabolism, i.e., processes that are defective in PD [17,18,20,21]. Notably, in this context, PINK1 plays a direct role in mitophagy [17,22]. In addition, the ER is responsible for properly folding newly synthesized proteins. When these proteins are folded incorrectly, they accumulate in the ER, leading to ER stress and activating the unfolded protein response (UPR), both of which are related to PD [23,24]. Finally, mitochondrial dysfunction and a defective sphingolipid metabolism have been identified to play a role in the pathogenesis of PD [25,26,27]. Thus, elements affecting the penetrance of PD are most likely involved in these cellular processes. For example, a recent study on homozygous twins discordant for idiopathic PD links mitochondrial integrity to altered disease penetrance [28]. However, the underlying mechanisms remain elusive. Hence, additional studies are required to understand the mechanisms that result in reduced penetrance.

Studies in *Drosophila melanogaster* have significantly contributed to our current knowledge of the mechanisms implicated in PD [29]. Research using the fruit fly was the first to highlight a common pathway between Pink1 and Parkin [30,31]. This finding was confirmed in human cellular systems [32,33]. Interestingly, in *Drosophila melanogaster*, reduced penetrance is observed concerning the flying phenotype in *pink1*-mutant flies. Previous studies show that the loss of Pink1 in flies results in a variable flying ability with a mean of around 10% [8,27,34], even though all these flies carry the same loss of function deletion in Pink1. Thus, the fruit fly is a suitable model to study mechanisms underlying altered penetrance.

Here, we performed a transcriptome analysis in *pink1*-mutant flies to identify alterations in RNA expression levels that affect the flying ability. The most significant upregulation of biological processes was observed primarily in pathways associated with transcriptional and translational activities and endoplasmic reticulum (ER) regulation. Conversely, downregulated pathways were linked to flagellated movement and microtubule organization. In addition, four genes were tested for validation, of which two, zonda (Zda) and windbeutel (wbl), were confirmed to be beneficial and involved in autophagy and ER stress, supporting a role of reduced penetrance for these cellular mechanisms.

## 2. Results

### 2.1. Lack of Flying Ability of Pink1-Deficient Flies Shows a Pattern of Reduced Penetrance

**Figure 1:**
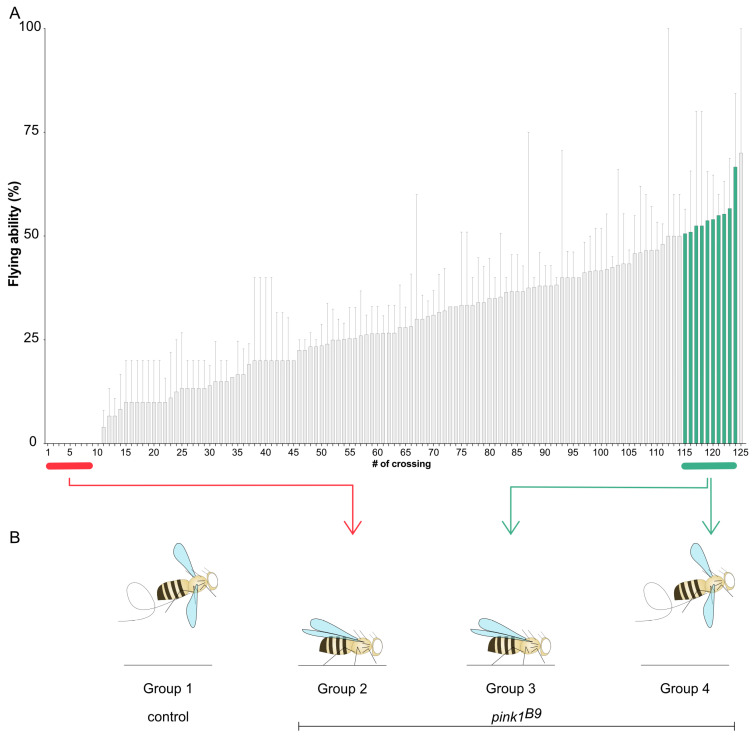
Reduced penetrance of the lack of flying ability of Pink1-deficient flies. (**A**) The flying ability of the offspring of 125 parent pairs of *pink1*-mutant flies. (**B**) Scheme to identify the different groups on which RNA sequencing analyses were performed. Data are percentages with sem; n > 5.

The average flying ability of general *pink1^B9^*-mutant fly stock is around ten percent [8], suggesting that a protective mechanism allows these ten percent of flies to fly. To analyze genetic factors that play a role in this reduced penetrance, we set up a group of 200 parent pairs consisting of one male and one virgin female fly to reduce the genetic variability of the offspring. One hundred and twenty-five parent pairs resulted in Pink1-deficient offspring and were tested for their flying ability. The offspring showed substantial variability in the ability to fly, ranging from a complete inability to fly to a flying ability of 60% (Figure 1A). Although we reduced the genetic variability by combining only one male and one female, the offspring of these parent pairs are not genetically identical, explaining that not all offspring of a specific parent pair display the same flying ability. This provides a direct tool to analyze the existing reduced penetrance, and our screen allows for the identification of genetic factors impacting the penetrance of the flying phenotype.

### 2.2. RNA Sequencing Analysis Identifies Genes Involved in Reduced Penetrance

To identify genes that play a role in reduced penetrance, we performed RNA sequencing analyses on four different groups (Figure 1B): 1. control flies (*pink1^RV^*); 2. *pink1^B9^*-mutant flies, of which none of the offspring could fly; 3. *pink1^B9^*-mutant flies that could not fly; 4. ‘siblings’ of group 3 that could fly (group 4). Thus, groups 3 and 4 were offspring of the same parent pair and had the lowest genetic variability; hence, differential RNA expression between those two groups may be the most relevant in the context of reduced penetrance.

**Figure 2:**
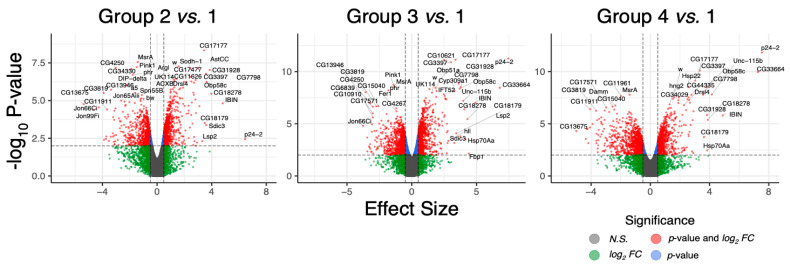
Volcano plots showing groupwise differentially regulated genes. The x- and y-axes denote the effect size (beta-value) and negative log_10_ values of the *p*-value corrected for multiple testing, respectively. Values are based on the likelihood-ratio test using sleuth. Significantly regulated genes (adj. *p*-value < 0.05; |effect size| > 1.0) are shown in red and annotated with their gene symbol.

In our group analysis, genes were designated differentially expressed with an absolute effect size (beta-value) > 1 and an adjusted *p*-value < 0.05. Notably, we identified 350, 611, and 650 differentially expressed genes (DEGs) when comparing the control group against groups two, three, and four, respectively (cf. volcano plots in Figure 2). When considering genes with human orthologs only, the gene count was substantially reduced, resulting in 144, 226, and 261 DEGs for the control comparison against groups two, three, and four.

**Figure 3:**
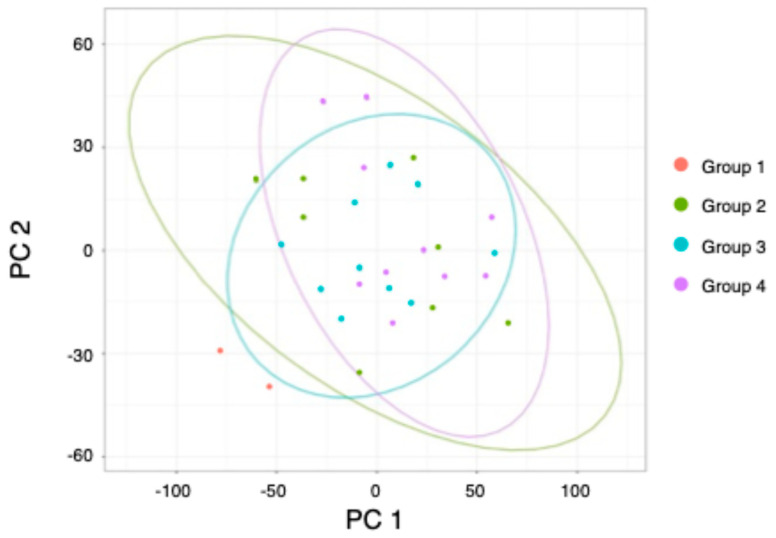
Principal component analysis showing the first two principal components (PC1/2) using TPM values of the 2000 most variable genes. Colors represent the different groups, as shown in the legend, while the ellipses encapsulating the groups assume a multivariate t-distribution of the data.

While we found many differentially regulated genes when comparing groups 2–4 to group 1, few significant DEGs were identified when comparing groups 2–4 to each other. A principal component analysis (PCA) further confirms gene expression similarity among these groups (Figure 3). Using the 2000 most variable transcripts based on transcripts per million (TPM) values across all samples, the three mutated groups clustered closely, as depicted by the ellipses encircling each group. Only the samples from group 1 are positioned further apart (red dots, Figure 3). Overall, we identified 12 genes that displayed significant RNA expression differences between groups 3 and 4, i.e., Pink1-deficient flies that cannot fly vs. those that can fly coming from the same parent pair.

To assess the penetrance of the loss of Pink1, we examined genes differentially regulated in controls and mutants that retained their flying ability compared to non-flying mutants. The comparison of the established groups (Flying, MutatedFlying, and Mutant) with the multiple regression model from limma revealed 3396 genes that were significantly reduced to 1692 genes when filtered for human orthologs. Additionally, we refined our gene selection criteria to include only those that exhibited either up- or down-regulation in both groups when compared to mutated, non-flying fruit flies. To enhance specificity and eliminate non-penetrance-related noise, we restricted the magnitude of expression changes between these groups to a factor of 3. Consequently, we successfully identified 124 genes, as detailed in Table S2.

### 2.3. Validation of the Protective Effects of Single Hits

**Figure 4:**
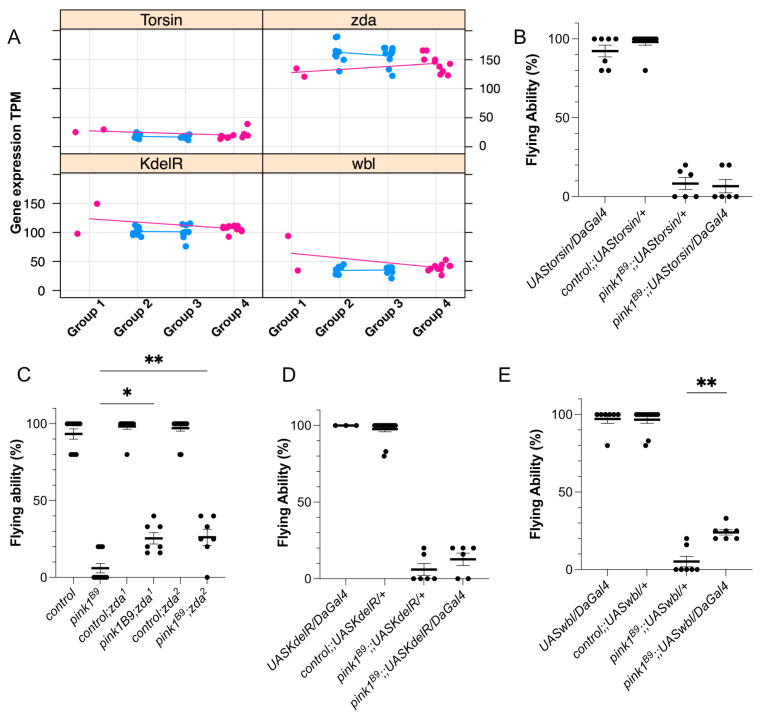
Validation of the protective effects of selected positive hits. (**A**) Gene expression of relevant hits from the multiple regression analysis. Normalized gene expression is plotted for the different groups, where the points for control and the mutated flyable offspring are colored in pink, while the data points of parents and non-flyable offspring are colored in blue. The genes selected showed annealing of mean gene expression for flyable offspring to the control level, while the mutated group remained higher/lower than the expression of the controls. (**B**–**E**) Flying ability of over-expression using the ubiquitous driver Daughterless Gal4 (DaGal4) of Torsin (**B**), KdelR (**D**), and wbl (**E**) or heterozygous loss of Zda (**C**) in a *pink1*-mutant background to validate the identified hits. Data are single data points of percentages with sem; n ≥ 3. *: *p* < 0.05; **: *p* < 0.01.

Pathway analyses revealed a significant enrichment of transcriptional and translational activities and regulation of (endo)membranes and ER, as well as a decrease in pathways related to flagellated movement and microtubule organization. The ER is known to be linked to neurodegeneration [35]; however, the underlying processes are not fully understood. Hence, we selected four ER-linked genes, which were also significant in the ANOVA test and that were readily available for testing: Torsin, KdelR, and winbeutel (wbl), which showed increased differential expression levels, and zonda (Zda), which displayed lower differential expression levels (Figure 4A), approaching control expression levels. The overexpression of KdelR and Torsin in Pink1-deficient flies failed to elicit an effect on the flying ability of *pink1*-mutant flies (Figure 4B,D), while the overexpression of wbl and two independent alleles of heterozygous loss of Zda improved the flying ability (Figure 4C,E).

## 3. Discussion

In this work, we showed the presence of reduced penetrance in Pink1-deficient flies and performed RNA expression analyses to elucidate possible mechanisms underlying this observation. Via gene ontology and pathway analyses, we identified several molecular mechanisms that appear to be involved in the penetrance of loss of Pink1, including ER-related activity. Finally, we confirmed a beneficial effect of lower Zda and higher wbl levels on the flying phenotype of *pink1*-mutant flies, suggesting that ER-linked autophagy and ER stress play an essential role in the penetrance of a PD-like phenotype in our *Drosophila* model.

The low genetic variability between the parents and offspring groups is to be expected since we assume that the genetic background should still be relatively close to its progenitor individuals. Additionally, the offspring groups were sampled from the same time point, resulting in an even higher genetic resemblance. In an attempt to circumvent this, we decided that a multiple linear regression would be the most fitting model, only focusing on the flying ability of the different sample groups. This also enabled us to work with a rather small control group of two sequencing samples since limma is fit for handling small sample sizes, and the multiple linear regression grouped the controls with the flying mutants, allowing us to pinpoint potential genetic variables that could affect the penetrance of the flying phenotype. By applying our set criteria for candidate genes (relevant pathways, significant ANOVA hits, same direction of logFC, human ortholog), we identified 124 genes that approach control expression levels in our mutants. Most genes in Table S2 still need to be identified, which makes them potential candidates for the modulation of penetrance that is challenging to evaluate experimentally.

The comparison of DEGs between groups 3 (*pink1*-mutant flies that cannot fly) and 4 (*pink1*-mutant flies that can fly and are ‘siblings’ of group 3) revealed twelve candidate genes to be altered, suggesting that these genes affect the reduced penetrance of the flying ability. Nonetheless, we did not validate these genes due to the assumption that it is unlikely that one gene can regulate penetrance. Instead, (minor) alterations in biological processes or cellular mechanisms are more likely to affect the penetrance. One of the identified pathways entails transcriptional and translational activities. Previously, PARIS, a Parkin-interacting substrate, was found to be accumulating following a loss of Parkin, exerting a detrimental effect on rRNA transcription [36].

Furthermore, the structure and function of mitochondrial ribosomes play a crucial role in determining oxidative phosphorylation efficiency. This feature is commonly affected in PD [37], providing possible explanations of how these biological processes impact the penetrance. Microtubule organization, another identified pathway, is key for the structural support of the cytoskeleton, facilitates intracellular transport, and contributes to the organization of cellular organelles. The dysfunction of microtubules is strongly associated with PD; however, it remains unclear whether this dysfunction is a cause or a consequence of PD [38]. Our data support that these pathways are involved in the reduced penetrance of the Pink1-deficient flying phenotype; however, how and to what extent remain elusive.

The ER is another biological process that plays a role in the reduced penetrance of the ability of *pink1*-mutant flies to fly. The ER has apparent links to neurodegeneration via its function in protein quality control and folding, mitophagy, and lipid homeostasis [21,23,24]. Poor function or dysregulation of one of these processes induces ER stress and can lead to neuronal cell death and, thus, neurodegeneration, such as Alzheimer’s disease and PD [35]. Therefore, we tested four genes, each showing significant transcriptional alterations separately, and will discuss these candidates in more detail below.

One of the proteins involved in ER stress is KDELR. KDELR plays key roles in maintaining ER homeostasis, protein trafficking, ER stress regulation, and autophagy [39,40]. Interestingly, KDELR is upregulated upon ER stress, which is also observed in PD [35,39,41], resulting in autophagy induction [39]. Furthermore, autophagy is a process that is affected in (Pink1-related) PD [25,27,42], suggesting that KDELR upregulation can be beneficial to overcome defective autophagy upon PD. This is in line with the findings of our initial screen, in which higher expression levels improve the flying ability. However, we were not able to reproduce these findings by overexpressing KDELR. One possible explanation could lie in the limitations of the applied GAL4/UAS system. This system overexpresses a protein beyond physiological levels [43] and is temperature-sensitive [44]. In our experimental setup, we grew the flies at 25 °C, which supports an efficient activation of the GAL4/UAS system, while the increased expression levels identified are only tenfold higher. These data suggest that too much KDELR abolishes the positive effect in *pink1*-mutant flies and that there is a small therapeutic window.

Interestingly, wbl is linked to KDELR, further supporting the involvement of KDELR and its processes in the penetrance of PD symptoms. Wbl is a protein disulfide isomerase (PDI)-related chaperone [45,46] that contains a C-terminal ER retention motif KDEL [47]. This motif interacts with KDELR to retrieve these ER chaperones from the Golgi [48,49]. The human ortholog for wbl is the endoplasmic reticulum protein of 29 kDA (ERp29), which is ubiquitously expressed [49]. ERp29 binds to misfolded proteins to facilitate proper folding or target them for degradation. Similar to the KDELR, ERp29 is upregulated upon ER stress to mitigate the accumulation of misfolded proteins and reduce cellular damage. Dysregulation of ERp29 is linked to several cancers and may serve as a biomarker for the progression of the tumor [50]. Furthermore, ERp29 is elevated following a dopamine exposure-related PD model, suggesting that ER stress occurs in the early stages of PD [51]. Our data show that increased ERp29 levels benefit the Pink1-deficient flying phenotype, supporting the notion that ER stress and its associated elevated ER proteins are a compensatory mechanism in this Pink1-related PD model.

TorsinA, the human ortholog for Torsin, is an AAA ATPase localized to the ER and the nuclear envelope. TorsinA maintains proteostasis and lipid metabolism and regulates intracellular vesicle trafficking [52,53,54]. TorsinA is encoded by the *TOR1A* gene, a mutation that causes early-onset dystonia [55]. Furthermore, impaired protein folding and increased ER stress due to TorsinA dysfunction [56] may contribute to neurodegeneration. Previously, Torsin has been linked to PD via its presence in the Lewy bodies [57,58]. Furthermore, in *C*. *elegans*, increased Torsin exerts a protective effect in a 6-hydroxydopamine (6-OHDA) PD model [59], supporting the findings from our screen where higher Torsin expression is beneficial for the flying phenotype in Pink1-deficient flies. However, via the GAL4/UAS system, we were unable to confirm these results, while overexpression in *C. elegans* did lead to a rescue. Unfortunately, these data are difficult to directly compare due to the unknown expression efficiency and the difference in the human disease and phenotypes observed in animal models. Hence, while increased Torsin levels have the potential to be protective, further investigations are required to determine the exact effect of Torsin, its relationship to the ER function, and its effect on PD.

Zda is an immunophilin localized at the ER. Zda is required for starvation-induced autophagy and interacts with the autophagy genes Atg1, Atg6, and Vps34 (vacuolar sorting protein) to activate the autophagy-specific activation of Vps34 and thus the initial stages of autophagy [60]. The human ortholog of Zda is FKBP8, an anti-apoptotic protein that assists in protein folding and the stabilization of proteins. FKBP8 is implicated in cardiovascular diseases, and studies in mammals suggest developmental abnormalities [61,62]. Furthermore, FKBP8 contains a microtubule-associated protein 1 light chain 3 (LC3)-interacting region (LIR) motif. It recruits LC3 to the mitochondria to induce Parkin-independent mitophagy while escaping degradation [63]. Autophagy or mitochondrial-specific mitophagy are processes that are affected in PD. Namely, PINK1 functions in mitophagy [25,27,32,64], suggesting that the beneficial effect of Zda lies in its mitophagy function. Previous studies showed that lowering mitophagy activity is beneficial for PINK1-dependent PD [27,42]. Thus, decreased Zda expression provokes lower mitophagy levels in an attempt to improve the *pink1*-mutant flying phenotype. In addition, under stress conditions, FKBP8 equally plays a role in mitochondrial fragmentation. Via its LIR-motif-like sequence (LIRL), FKBP8 binds to OPA1, mediating fragmentation and enabling mitophagy [65]. Reversely, the knockdown of FKBP8 enlarges the mitochondria to form its natural tubular network. Interestingly, the loss of Pink1 induces mitochondrial accumulation and loss of the tubular network [8,31]. Thus, decreased Zda levels could induce a more tubular mitochondrial network, allowing the mitochondria to function more efficiently. Therefore, the beneficial role of reducing Zda levels may extend to a dual role in inhibiting mitophagy and mitochondrial dynamics.

This study is the first to identify possible cellular mechanisms involved in the penetrance of the flying ability in a PD model. The fly model proved to be of excellent value for investigating and identifying biological processes involved in reduced penetrance. Nonetheless, we were not able to validate all the selected candidates, possibly due to the limitations of the applied genetic tools. Lowering the incubation temperature ensures a less efficient UAS-Gal4 system and, hence, lowers the UAS lines’ overexpression. However, as the alterations in RNA expression are rather minor, the reduced overexpression would still be too high to mimic the results of RNA expression. Alternatively, a genomic construct could be created to overcome this.

Furthermore, with this setup, we could not validate if these biological processes equally affect other phenotypes in *pink1*-mutant flies, nor did we test their relevance to patients. For this validation in a clinical context, our data should first be tested in a higher species model, such as patient-derived dopaminergic neurons. In these cells, the effect of manipulation of the ER to influence ER stress can be assessed for its evolutionary conservation and to test if reducing ER stress is a valid therapeutic target to affect the penetrance of PD in as yet unaffected pathogenic variant carriers. In conclusion, our data further point to the ER implicated in PD and its penetrance in a genetic PD fly model that needs further validation in a patient-relevant context. In addition, further investigation into the exact underlying mechanism is necessary so that this pathway can be modulated to alter the penetrance for PD.

## 4. Materials and Methods

### 4.1. Fly Genetics

*w pink1^B9^* null mutants and controls (*w pink1^RV^*) were kindly provided by Jeehye Park and Jongkyeong Chung (Korea Advanced Institute of Science and Technology) [30]. y[1] w[*]; M{RFP[3xP3.PB] w[+mC]=UAS-KDELR-RFP}ZH-22A (UASKdelR) were generously shared by the Katanaev laboratory (Department of Pharmacology and Toxicology, University of Lausanne) [66]. Two independent lines of Zda (w1118; PBac{IT.GAL4}zda0818-G4 (Zda^1^) and y1 w67c23; P{EPgy2}zdaEY08359 (Zda^2^)) and *daughterless Gal4* (DaGal4) were purchased from the Bloomington Stock Center (Indianapolis, United States of America). M[UAS-Torsin.ORF.3xHA.GW]ZH-86Fb (UASTorsin) and M[UAS-Wbl.ORF.3xHA.GW] (UASWbl) were purchased from the FlyORF stock center (Zurich, Switzerland).

### 4.2. Flight Assay

One-day-old male flies were collected and tested for their flying ability. Flies were placed in an empty vial that was gently tapped. Flies able or unable to fly were scored one and zero, respectively [8]. For the sequencing analyses, the offspring of each parent pair that was able to fly (group 4) was separately collected from those that were not able to fly (group 3).

### 4.3. RNA Sequencing and Analyses

RNA sequencing was performed on four different groups. Group 1 is control flies (*pink1^RV^*), group 2 is *pink1^B9^*-mutant flies of which none of the offspring were able to fly, and groups 3 and 4 are *pink1^B9^*-mutant flies that are offspring from the same parent; however, in group 3, none of the flies were able to fly, while in group 4, all the flies were able to fly. RNA was isolated from five flies via a standard procedure (Qiagen, Venlo, The Netherlands), and RNA sequencing was performed by the genomics core UZ Leuven (Belgium) using an Illumina Hiseq system.

The fastq files underwent preprocessing using fastp (version 0.23.4) [67], wherein reads with less than 30% of bases possessing a quality PHRED score under 15 were selectively retained. Subsequently, reads were pseudoaligned to the *Drosophila melanogaster* genome assembly BDGP6.46 through kallisto (version 0.44.0). The alignment metrics are detailed in Table S1.

Differential expression and pathway analyses were executed in R (version 4.2.2), primarily employing sleuth (version 0.30.1) [68] or limma (version 3.54.2) [69]. Sleuth was utilized for straightforward group comparisons, while limma and its extension of ANOVA tests were applied for more intricate model computations. Gene annotation was derived from the genome-wide annotation for *Drosophila melanogaster* package org.Dm.eg.db (version 3.16.0), encompassing entries for HGNC and flybase nomenclature, GO terms, and Homo sapiens gene homology.

Expression data analysis with limma was performed on kallisto’s abundance levels, which were transformed into length-scaled count values using tximport (version 1.26.1) [70]. Pathway analysis was conducted using GAGE (version 2.48.0) [71], incorporating either the log_2_ fold changes from differential expression or sleuth’s beta values. We corrected differential gene expression and pathway significance for multiple testing using the BH correction. The cutoff for the adjusted *p*-value was set to 0.05 in all our analyses.

To investigate penetrance, we combined the sample groups into flying, mutated, and non-flying and looked for co-regulation between the two flying groups when compared to the mutated non-flying group with multiple comparison testing. Specifically, the Flying group consisted of samples from the controls (group 1) and the offspring with flying ability (group 4). The MutatedFlying group only encapsulated the flying offspring (group 4), while the Mutated group combined the samples from offspring that were unable to fly (group 2 and 3). Using this design, we calculated the DEGs between Flying versus Mutated and MutatedFlying versus Mutated and subsequently called the limma equivalent of an ANOVA and these multiple comparisons.

## Data Availability

The data has been uploaded to Gene Expression Omnibus (GEO) and is available under the accession ID GSE287758.

## Supplementary Materials

The supporting information can be downloaded at: https://www.mdpi.com/article/10.3390/ijms26030979/s1.
